# Haptic Fidelity: The Game Changer in Surgical Simulators for the Next Decade?

**DOI:** 10.3389/fonc.2021.713343

**Published:** 2021-08-11

**Authors:** Valentin Favier, Gérard Subsol, Martha Duraes, Guillaume Captier, Patrice Gallet

**Affiliations:** ^1^Department of Otolaryngology-Head and Neck Surgery, Gui de Chauliac Hospital, University Hospital of Montpellier, Montpellier, France; ^2^Laboratory of Anatomy of Montpellier, Faculty of Medicine, Univ. Montpellier, Montpellier, France; ^3^Research-team ICAR, Laboratory of Computer Science, Robotics and Microelectronics of Montpellier (LIRMM), Univ. Montpellier, French National Centre for Scientific Research (CNRS), Montpellier, France; ^4^Department of Otolaryngology-Head and Neck Surgery, Brabois Hospital, University Regional Hospital of Nancy, Lorraine University, Vandoeuvre-lès-Nancy, France; ^5^Virtual Hospital of Lorraine, University of Lorraine, Vandoeuvre-lès-Nancy, France; ^6^NGERE, INSERM U1256 lab, University of Lorraine, Vandoeuvre-lès-Nancy, France

**Keywords:** surgical simulation, haptics, fidelity, simulator assessment, surgical education

## Introduction

Over the last 20 years, surgical simulation has evolved tremendously from bench models to “high-fidelity” virtual reality surgical simulators. The main objective of these simulators is to acquire the technical skills to be transferred to the operating room without any risk for patients. In this intent, both simulator and the progression of the simulation training process (1) must be validated and follow the standards (2). Nevertheless, a facet of surgical simulation lacks a deep consideration: the haptic feedback, which is essential in most surgical procedures, is rarely assessed and the place of haptic fidelity is unclear (3). Then, how can we determine the place of haptic fidelity in surgical simulation training?

## Fidelity and Simulation in Surgery

The definition of “fidelity” in healthcare simulation remains a major matter of debate. Fidelity is, in essence, a multifactorial concept (4). It refers to sensory resemblance (auditory, visual, tactile) as well as functional resemblance and, therefore, depends on the context and learning objectives. In surgical simulation, fidelity has too often been reduced to “face validity” (i.e., the simulator “looks like” the reality) and even more reduced to visual resemblance. The underlying concepts of “face validity”—i.e., the perception of the user which contributes to simulator credibility, adhesion to it, and enhance information retention and transfer to practice—are relevant but a visual resemblance (closeness to the shape and color of the anatomical structures) is not sufficient to assess a surgical simulator. This is why “face validity” is not part of the Standards for Educational and Psychological Tests and Manuals (2) even if it continues to be wrongly used.

In fact, Paige and Morin (4) defined three dimensions of fidelity and proposed a “fidelity matrix” based on the following:

1. physical (or engineering) fidelity (of the equipment and environment);
2. psychological fidelity, which ensures that the trainee is engaged in the simulation. It characterizes the extent to which events and scenarios reflect real situations and the extent to which the simulator provides realistic responses to the actions of the learners; and
3. conceptual fidelity, which ensures that the scenario makes sense and corresponds to the human conceptual mode of thinking, such as problem solving or decision-making.

Each dimension is independent and has to be adapted to the context and task to be simulated. Following these concepts, Hamstra et al. (5) proposed to distinguish the “physical resemblance” from the “functional task alignment” (FTA) (i.e., how much a simulator functions like the reality in response to the actions of a user). They advocated that the conception and design of simulators should follow the FTA analysis to functionally represent a patient in response to the physical action of the task, rather than seeking to achieve a physical resemblance. Following this definition, the simulator fidelity is achieved when the simulator functional parts are in congruence with the learning objectives.

## Toward a Better Evaluation of Haptic Fidelity in Surgical Simulation

One of the components of the physical resemblance is the haptic fidelity (i.e., the perception by touch and proprioception). Haptic skills are crucial to learning surgery: it is of utmost importance for a surgeon to learn how to handle tissues safely, how to “feel” the dissection plan, and how to palpate a tumor. These haptic skills are essential for a safe use of surgical instruments, spatial representation, understanding of tumor relationships and limits, and evaluation of surgical risks at each step of a procedure. In surgery, the risk of a regular training with a simulator lacking realistic haptic rendering is to provide a negative transfer in the operating theater, where the learner might apply dangerous forces which is possibly difficult to untrain later (6). Therefore, the haptic fidelity assessment of surgical simulators is essential and should be taken into consideration from the beginning of the design of the simulators, according to the simulated tasks (in agreement with FTA). For example, to simulate a realistic neurovascular dissection, the simulated tissues should be adequately adherent to each other and have a consistent physical behavior to experience which forces should be applied on anatomical structures. Haptic feedback is all the more important in endoscopic surgery (7), in which surgeons are proceeding without directly seeing their hands, only with the visual support of a two-dimensional display, and in which haptic feedback provides crucial information for decision-making and movement planning. Thus, the study of haptics is of growing interest especially for virtual reality surgical simulation which deals with the same issues (8). It was shown that haptic feedback may play an important role in motor skills acquisition (9), but haptic skills are difficult to teach in an objective and standardized manner. Therefore, research on procedural simulation should focus on biomechanical characterization of the tissues to be simulated, to select suitable synthetic materials for physical simulators and improve the haptic feedback of virtual reality simulators. Most virtual reality systems provide force feedback only for anatomical structures that have been segmented and assigned properties, but such biomechanical data are often missing (3).

A recent review of the literature (8) highlighted “the inconsistency and paucity of current evidence with regard to haptics and its validity and evidence of its value in [surgical] training”. Few authors actually measured the applied forces or the precision of movements during surgical procedures which give quantitative parameters that could be used for haptics application (10–12). Moreover, these well-designed studies are often published in biomedical engineering journals and may be poorly disseminated within the medical community. Finally, the assessment of haptic fidelity on surgical simulators is often qualitative, provided by users or designers, and usually compared to “reality” using binary questionnaires or Likert-type scales. As an illustration, Chan et al. (13), who conducted a very substantial work on modeling and virtual rendering for temporal bone surgery simulation, evaluated the “high-fidelity” on visual appearance (comparison with videos) only rather than on objective parameters (such as the amount of bone removed or the applied forces). We believe that this approach is insufficient to create and validate “high-fidelity” haptic procedural simulators. It is indeed with a better knowledge of the biomechanical characteristics and through the objective evaluation of these parameters that the evaluation of haptic fidelity would be more relevant than questionnaires specific to each simulator designer/evaluator.

## Defining Haptic Fidelity Levels Adapted to Surgical Tasks

The FTA theory (5) highlights the need to think about the objectives of the learners before designing a simulator. Thus, we advocate for a more robust method of classification of the level of fidelity, in accordance with the learning objectives (FTA-based approach).

**Figure 1:**
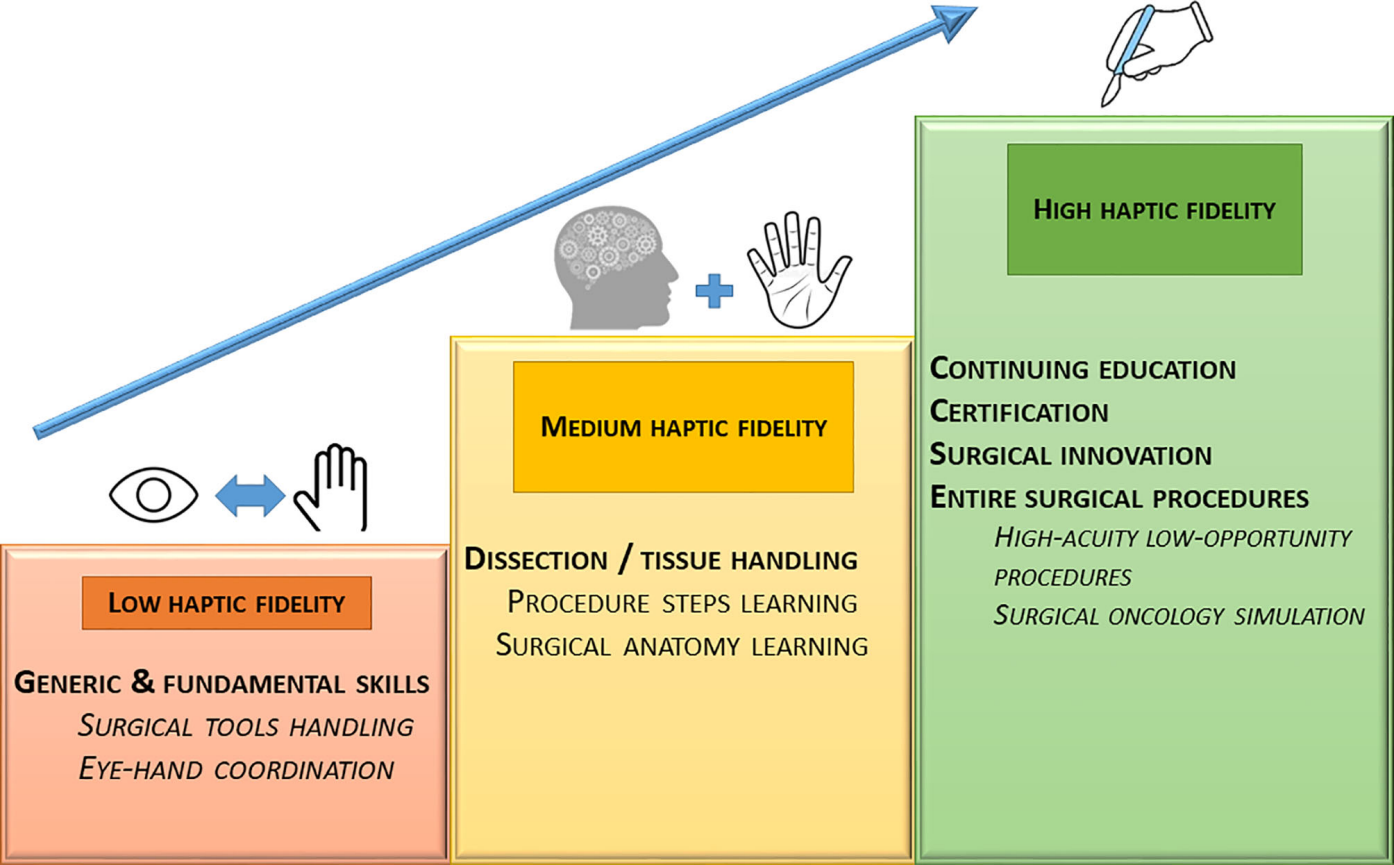
Proposed levels of haptic fidelity and corresponding skills to learn.

The first step is to define which level of haptic fidelity is required according to the surgical skill to learn. Indeed, basic technical skills (e.g., navigation with an endoscope) do not always require high haptic fidelity levels, in contrast to some advanced skills (e.g., neurovascular dissection). We propose to define three levels of haptic fidelity (**Figure 1**). Simulators with a low haptic accuracy level (i) could be used to learn basic manipulations of surgical materials or instruments and to increase the eye-hand coordination. Simulators with a medium haptic accuracy level (ii) could be used to become familiar with the surgical procedures (e.g., learning procedure steps). Finally, high haptic accuracy simulators (iii) may help in the acquisition of fine technical skills like tumor dissection. This distinction might help in undertaking a preliminary reflection on the essential aspects of the task to be simulated to choose the appropriate level of fidelity, before designing any training device or create a surgical curriculum.

The second step is to provide guidance on the haptic fidelity assessment. The experiment of Batteau et al. (14) showed that haptic recall consistency (i.e., how consistently a haptic experience can be recalled) can vary widely among individuals and may be independent of experience. This finding suggests that the method of using “expert” opinion to fine-tune haptic feedback in surgical simulators may be insufficiently reliable, as it depends on the ability of the experts to accurately recall and compare haptic events. In all other domains of training by simulation, efficacy and learning transfer can be assessed using Kirkpatrick’s pyramid model (15). Haptic rendering fidelity could then be adequately assessed according to a similar scheme in order to determine the value and level of a simulator, using three grades of accuracy: a) not proven accuracy, absence of assessment of the simulator haptic feedback; b) subjective accuracy, favorable opinion on the haptic accuracy provided by a panel of experts; and c) objective accuracy, proof of the haptic rendering accuracy through biomechanical measurements. According to Mahvash et al. (16) and, more recently, Lelevé et al. (17), biomechanical measurements and objective tests may include at least:

1. tissue basic physical (texture, shape, volume, weight, temperature) and mechanical properties (compressive, tensile, bending, or shear properties) measurements. These properties can easily be assessed by calibrated testing for both soft (18) and hard tissues (19). For instance, erroneous elastic properties of soft tissues could lead to an inadequate manipulation of tumors next to neurovascular structures leading to injuries; and
2. characterization of the tissue response to different surgical actions (scissor cutting, drilling, peeling, biting, twisting, etc.). This can be done by measuring forces applied on tissues with surgical instruments commonly used in the operating room (20–22). For instance, a synthetic plastic device perfectly mimicking a bone tissue as for mechanical resistance to compression might behave unrealistically with other actions like drilling (the plastic might melt with the drilling overheating) or twisting (with unrealistic fracture line, due to the orientation of resistance spans).

Assessing haptic rendering accuracy is an absolute requirement for simulators to be used for surgical training certification—a process by which individuals are recognized (or certified) as having demonstrated some level of knowledge and skill in some domain (2). This certification is already the norm in aviation where pilots must have accumulated a specific number of hours of flight training with validated simulators. This is not routinely done in surgery, but the society attitude toward surgical training will certainly lead to the requirement of an objective proof of the skills of the surgeon before being allowed to work with patients. Haptic fidelity can be the game changer of the decade.

## Author Contributions

All authors contributed to the article and approved the submitted version.

## Funding

The first author (VF) received the funding of the “Collège Français d’ORL et chirurgie cervico-faciale” for a scholarship from November 2019 to November 2020 to support his PhD thesis.

## Conflict of Interest

The authors declare that the research was conducted in the absence of any commercial or financial relationships that could be construed as a potential conflict of interest.

## Publisher’s Note

All claims expressed in this article are solely those of the authors and do not necessarily represent those of their affiliated organizations, or those of the publisher, the editors and the reviewers. Any product that may be evaluated in this article, or claim that may be made by its manufacturer, is not guaranteed or endorsed by the publisher.
